# Caring for Children With Medical Complexity in the Early COVID-19 Pandemic in an Ambulatory Primary Care Setting

**DOI:** 10.3389/fped.2022.813393

**Published:** 2022-02-09

**Authors:** Allison Driansky, Mariecel Pilapil, Kristina Bianco, Caren Steinway, Sheri Feigenbaum, Anna Yang, Sophia Jan

**Affiliations:** ^1^Steven and Alexandra Cohen Children's Medical Center, Pediatrics, New Hyde Park, NY, United States; ^2^Donald and Barbara Zucker School of Medicine at Hofstra, Northwell Health, Hempstead, NY, United States; ^3^South Shore University Hospital, Northwell Health, Bay Shore, NY, United States; ^4^Children's Hospital of Philadelphia, Philadelphia, PA, United States

**Keywords:** children with medical complexity, COVID-19, general pediatrics, complex care, caregivers

## Abstract

**Background:**

Children with medical complexity (CMC) have multiple chronic conditions and require a high level of coordinated healthcare. The risk of COVID-19 among CMC is unclear.

**Objectives:**

We aim to identify and describe the prevalence and experience of COVID-19 among CMC and their caregivers during the initial weeks of the COVID-19 pandemic in the NY metropolitan area.

**Methods:**

We performed a cross-sectional study of children enrolled in a structured clinical program for CMC at a large urban, academic general pediatrics practice in NY.

**Results:**

In our patient population (*n* = 132), 16 patients had a known exposure with parents being the most common exposure in 37.5% (*n* = 6). Two patients were hospitalized for COVID-19 while the remainder of the confirmed or suspected COVID-19 cases were managed as an outpatient.

**Conclusions:**

Common sources of COVID-19 exposure were family members and home care providers. Almost all of our patients experienced interruption of medical care including missed therapies and visits.

## Introduction

The COVID-19 pandemic caused by severe acute respiratory syndrome (SARS-CoV2) is a major health crisis affecting all aspects of society. While COVID-19 infection in the pediatric population appears to have a mild course in comparison to adults, children are still vulnerable to infection and complications (1, 2). Children with medical complexity (CMC) have multiple chronic conditions affecting many organ systems and require a high level of coordinated healthcare (3). Limited evidence to date has shown the most common comorbidity for critically ill children in pediatric intensive care units (PICUs) with COVID-19 is medical complexity (4). Although no studies have yet evaluated the effect of COVID-19 on the CMC population, CMC are generally at higher risk for developing respiratory complications due to factors such as chronic lung disease, impaired airway clearance, and ventilatory support at baseline, likely putting them at increased risk for morbidity and mortality from COVID-19. Additionally, CMC typically require multiple therapists, home nurses or home health aides, and case managers, increasing the number of individuals they are exposed to and subsequently their risk of exposure to COVID-19. Finally, missed primary care and sub-specialty appointments due to the COVID-19 pandemic may disproportionately affect CMC, who require significantly more health resources and coordinated healthcare than the general pediatric population.

The goal of this study was to describe the prevalence of and exposure to COVID-19 among CMC and their caregivers and barriers to healthcare that have affected CMC during the early days of the COVID-19 pandemic when stay at home orders were in effect.

## Methods

We performed a cross-sectional study of children (0–21 years) enrolled in a structured clinical program for children with medical complexity at a large urban, academic general pediatrics practice in New York City. All patients enrolled in this program were contacted via telemedicine (audio +/- video) between March 17, 2020 and May 1, 2020. Telemedicine visits were conducted using a predetermined checklist that addressed clinical concerns including COVID-19 and disruptions in care. Demographic data and information regarding COVID-19 status was collected through chart review in our ambulatory electronic medical record (EMR). The study was submitted to Northwell Health's Institutional Review Board (IRB) and was considered exempt.

## Results

A total of 132 patients were enrolled in the clinical program for CMC at the time of data collection (Table 1). In our population, 28.8% were Black or African American and 23.5% had a preferred language other than English. Most patients (87.9%) were publicly insured. The most common chronic conditions were: neurologic or developmental disability (81.8%), chronic lung disease (41.7%), and cardiac disease (32.6%). Patients had an average number of 2.2 chronic conditions. The majority of patients were technology dependent, with 56.8% requiring enteral feeding and 35.6% having respiratory support (tracheostomy/ventilator or non-invasive positive pressure ventilation).

**Table 1:** Demographic and clinical features of patient population.

	**Total (*n =* 132)**	**No COVID exposures (*n =* 116)**	**Known COVID exposure (*n =* 16)**	***P*-value**
**Demographics**				
Age (months, mean) (range)	72.5 (5–121)	73.0 (5–121)	68.9 (15–117)	0.36
Gender (*n*, %)				
Male Female	81 (61.3) 51 (38.6)	69 (59.5) 47 (40.5)	12 (75.0) 4 (25.0)	0.23
Race (*n*, %)				
White Asian Black or African American American Indian/Alaskan Native Other Unknown	12 (9.0) 23 (17.4) 38 (28.8) 1 (0.7) 50 (37.9) 7 (5.3)	12 (10.3) 21 (18.1) 32 (27.6) 1 (0.9) 43 (37.1) 6 (5.2)	0 (0) 2 (12.5) 6 (37.5) 0 (0) 7 (43.8) 1 (6.2)	0.75
Ethnicity				
Not Hispanic or Latino Hispanic or Latino Unknown	80 (60.6) 44 (33.3) 8 (6.1)	72 (62.1) 36 (31.0) 8 (6.9)	8 (50) 8 (50) 0 (0)	0.23
Preferred Language				
English Spanish Chinese	102 (77.3) 29 (22.0) 1 (0.76)	89 (76.7) 27 (23.3) 0 (0)	13 (81.3) 2 (12.5) 1 (6.2)	**0.02**
Insurance type				
Public (Medicaid/CHIP) Private Other	115 (87.1) 16 (12.1) 1 (0.01)	103 (89.7) 12 (10.3) 1 (0.01)	12 (75.0) 4 (25.0) 0 (0)	0.23
**Clinical features**				
Chronic conditions				
Neurologic or developmental disability Chronic lung disease Cardiac disease	108 (81.8) 55 (41.7) 43 (32.6)	94 (81.0) 47 (40.5) 39 (33.6)	14 (87.5) 8 (50.0) 4 (25.0)	0.53 0.47 0.49
Technology dependence				
Oxygen/Trach/Ventilator Nasogastric/Enteral feeding tube	47 (35.6) 75 (56.8)	41 (35.3) 66 (56.9)	6 (37.5) 9 (56.3)	0.87 0.96
^#^ of active medications (mean)	6.9	6.8	7.8	0.48
^#^ of specialists seen (mean)	5.2	5.1	5.4	0.61
^#^ of hospitalizations (past 1 year)	1.9	1.9	2.25	0.56
Total length of stay (LOS) (days) (past 1 year)	12.7	13.6	6.4	0.36
Ancillary care services (*n*, %)				
Home nursing PT/OT/Speech therapy Health home	49 (37.1) 112 (84.8) 57 (43.2)	44 (37.9) 99 (85.3) 53 (37.1)	5 (31.2) 13 (81.3) 4 (25)	0.60 0.67 0.12
**Ambulatory care**				
Contact with clinical team				
Patients who engaged in telemedicine (*n*, %) Mean # of telephonic encounters Mean # of telemedicine visits	48 (36.4) 1.36 1.02	40 (34.5) 1.10 0.86	8 (50.0) 3.19 2	0.23 ** <0.005** 0.06
Delayed/Canceled In-Person Visits (*n*, %)				
Primary care Subspecialty	31 (23.5) 73 (55.3)	29 (25.0) 62 (53.4)	2 (12.5) 11 (68.9)	0.27 0.25
Postponement of care (*n*, %)				
Vaccines Elective surgical procedures Routine dental care Routine eye care Routine laboratory studies	8 (6.1) 4 (3.0) 3 (2.3) 10 (7.6) 1 (0.8)	6 (5.2) 4 (3.4) 3 (2.6) 8 (6.9) 1 (0.9)	2 (12.5) 0 (0) 0 (0) 2 (12.5) 0 (0)	0.25 0.45 0.52 0.43 0.71
Interruption of home care services (*n*, %)				
Nursing PT/OT/Speech therapy	7 (5.3) 14 (10.6)	6 (5.2) 13 (11.2)	1 (6.3) 1 (6.3)	0.86 0.55
Difficulty obtaining supplies (*n*, %)				
Routine medications or supplies Enteral tube feedings	9 (6.8) 10 (7.6)	8 (6.9) 9 (7.8)	1 (6.3) 1 (6.3)	0.92 0.71

# *means “number”*.

Within this patient population, 36 patients developed symptoms associated with COVID-19. Of those, 11 had a known COVID-19 exposure. An additional 5 patients had a known exposure but did not develop symptoms. The most common source of exposure was a parent, followed by in-home care providers (home health aide, home nurse, or therapist) (Table 2). The most common symptoms among this cohort of 36 symptomatic patients were fever, cough, rhinorrhea, and shortness of breath. There were no differences in demographic or clinical characteristics between those with and without a COVID-19 exposure. Two patients were hospitalized for COVID-19. One patient experienced a heart failure exacerbation but did not require any respiratory support and was discharged home. The second patient experienced respiratory failure and eventually died. The remainder of patients with clinical symptoms of COVID-19 (both confirmed and suspected) were managed as an outpatient.

**Table 2:** COVID status of children with medical complexity.

	**Total *n =* 35 (*n*, %)**
Most commonly reported COVID symptoms	
Fever	20 (57.1)
Cough	13 (37.1)
Rhinorrhea/Nasal congestion	6 (17.1)
Shortness of breath/Difficulty breathing	4 (11.4)
Diagnostic testing (*n*, %)	
Confirmed (via nasal swab testing)	5 (14.3)
Required hospitalization (*n*, %)	2 (5.6)

## Discussion

This study provides a cross-sectional description of the characteristics and short term outcomes of the early phases of the COVID-19 pandemic on an ambulatory, medically complex, pediatric population in New York City during stay at home orders. Surprisingly, very few of the medically complex patients required hospitalization due to COVID-19 infection. This is in line with the larger body of research showing a milder course and generally good outcomes for COVID-19 infection in the pediatric population (1, 2, 4, 5). Despite this, pre-existing medical comorbidities do seem to play an important role for children that are hospitalized with COVID-19, particularly those who are critically ill (5, 6). One potential protective factor may be the role of the state-wide stay at home orders in the clinical experience of COVID-19 on our CMC. In addition, family caregivers implemented mitigation techniques such as foregoing home services and assuming full-time roles as home nurses and nursing assistants for their children (7). Without this mandate and the diligent safety protocols many families instituted, this cohort may have been more at risk for contracting COVID-19. Furthermore, COVID-19 vaccination is another important mitigation technique. Experts have advocated for prioritizing CMC and their caregivers (given their role as health care personnel) for COVID-19 vaccination and the need to ensure access to vaccination for this population (7, 8). The data described in this study was collected prior to widespread availability of COVID-19 vaccination, but future studies should examine vaccine uptake and its potential protective effects in this population.

CMC represent an important subset of pediatric patients that are susceptible not just to the primary complications of COVID-19 but also the secondary effects of the pandemic (9). Many of the public health measures put into place during the pandemic (social distancing, school closures, reduction in non-urgent health care visits) had unique consequences for CMC. Almost all our patients had medical care that was affected by the COVID-19 pandemic, with interruption of therapies and missed visits being the most common. This likely affects the general pediatric population as well, but may be more impactful for CMC.

The direct and indirect complications of COVID-19 may have been partly attenuated in our study by regularly scheduled, proactive, telephonic outreach to every member of this cohort. Widespread adoption and expansion of telemedicine during the COVID-19 pandemic has shown promise in managing both acute and chronic illness and providing routine care and therapies for patients (10–12). Telemedicine remains a powerful and promising tool to lessen care gaps and access issues.

CMC are especially susceptible to the direct and indirect effects of COVID-19 due to their reliance on a large interdisciplinary, interprofessional care team. During the pandemic, care teams have had to adapt to continue to meet the needs of CMC. For example, medical teams in the pediatric intensive care unit have incorporated telemedicine and virtual platforms to maintain multi-disciplinary care discussions to continue caring for patients (13). When our patients were exposed to COVID-19, the most common sources were family members and in-home care providers. Many parents of this medically complex patient cohort reported removing their children from in-person school environments or not allowing in-home care providers (i.e., care managers, therapists, nurses) into the home during that period in order to limit potential exposures. This is reflected in published data that showed a decrease of enrollment in home visiting services by 33–36% during the COVID pandemic (14). For CMC, the likelihood of COVID-19 exposure early in the pandemic reflected trends in the general population, most notably higher exposure risk in non-White racial groups, though not statistically significant in our analysis. This suggests that racial disparities in exposure can be detected even in small cohorts of children with CMC.

There are several limitations to this study. First, the described patient population was limited to CMC followed in one large academic pediatric practice. Additionally, during the early COVID-19 pandemic, testing in our region was primarily available only to hospitalized patients, likely underestimating the observed number of COVID-19 positive patients in our ambulatory practice. Furthermore, data in this study was collected through telemedicine encounters with patients and caregivers, and is therefore subject to recall bias and parental interpretation.

Despite its limitations, this study has several important implications. First, given the high rates of missed care, healthcare providers should focus on minimizing gaps in care for CMC with proactive outreach during the ongoing COVID-19 pandemic and future outbreaks. Second, given the high risk of exposure in parents of CMC, this study suggests the need to identify and train backup caregivers in case a primary caregiver becomes ill. Third, caregivers should consider the need to balance the risk of exposure from in-home care services with the necessity of the service provided.

Given the wide availability and feasibility of using telemedicine in patients with CMC (15), future directions should focus on expanding telemedicine to bridge care gaps for families. Furthermore, there is a distinct need to organize support for backup caregivers who may be inexperienced. Finally, pediatricians should deliver support for caregivers, who may be strained taking on more roles with limited resources in a pandemic setting.

This study represents the early effects of the COVID-19 pandemic on the CMC population. The emergence of new variants including delta and omicron has led to a rise in pediatric cases and therefore will have implications on CMC, who are more susceptible to progression to severe disease. It is essential that future studies be conducted to determine the ongoing threat of COVID-19 on this vulnerable population.

## Data Availability Statement

The data analyzed in this study is subject to the following licenses/restrictions: Datasets include PHI. Requests to access these datasets should be directed to astakofsky@northwell.edu.

## Ethics Statement

The studies involving human participants were reviewed and approved by Human Research Protection Program (HRPP) Feinstein Institutes for Medical Research Northwell Health. Written informed consent from the participants' legal guardian/next of kin was not required to participate in this study in accordance with the national legislation and the institutional requirements.

## Author Contributions

AD, MP, KB, CS, SF, AY, and SJ were involved in the planning, data collection, and authorship of this paper. All authors contributed to the article and approved the submitted version.

## Conflict of Interest

The authors declare that the research was conducted in the absence of any commercial or financial relationships that could be construed as a potential conflict of interest.

## Publisher's Note

All claims expressed in this article are solely those of the authors and do not necessarily represent those of their affiliated organizations, or those of the publisher, the editors and the reviewers. Any product that may be evaluated in this article, or claim that may be made by its manufacturer, is not guaranteed or endorsed by the publisher.
